# Application of Activated Carbons Obtained from Polymer Waste for the Adsorption of Dyes from Aqueous Solutions

**DOI:** 10.3390/ma17030748

**Published:** 2024-02-04

**Authors:** Katarzyna Jedynak, Barbara Charmas

**Affiliations:** 1 Institute of Chemistry, Jan Kochanowski University, Uniwersytecka Str. 7, 25-406 Kielce, Poland; 2 Institute of Chemical Sciences, Faculty of Chemistry, Maria Curie-Sklodowska University, Maria Curie-Sklodowska Sq. 3, 20-031 Lublin, Poland

**Keywords:** activated carbons, polymer waste, physical activation, dye adsorption, physicochemical properties

## Abstract

Plastic waste disposal is a major environmental problem worldwide. One recycling method for polymeric materials is their conversion into carbon materials. Therefore, a process of obtaining activated carbons through the carbonization of waste CDs (as the selected carbon precursor) in an oxygen-free atmosphere, and then the physical activation of the obtained material with CO_2_, was developed. Dyes such as methylene blue (MB) and malachite green (MG) are commonly applied in industry, which contaminate the water environment to a large extent and have a harmful effect on living organisms; therefore, adsorption studies were carried out for these cationic dyes. The effects of the activation time on the physicochemical properties of the activated materials and the adsorption capacity of the dyes were investigated. The obtained microporous adsorbents were characterized by studying the porous structure based on low-temperature nitrogen adsorption/desorption, scanning electron microscopy (SEM-EDS), elemental analysis (CHNS), Raman spectroscopy, X-ray powder diffraction (XRD), infrared spectroscopy (ATR FT-IR), thermal analysis (TG, DTG, DTA), Boehm’s titration method, and pH_pzc_ (the point of zero charge) determination. Moreover, adsorption studies (equilibrium and kinetics) were carried out. The maximum adsorption capacities (*q_m exp_*) of MB and MG (349 mg g^−1^ and 274 mg g^−1^, respectively) were identified for the obtained material after 8 h of activation. The results show that the use of waste CDs as a carbon precursor facilitates the production of low-cost and effective adsorbents.

## 1. Introduction

The production, consumption, and generation rate of solid plastic waste constitute a serious problem for the environment [1,2]. To counteract this, waste recycling can be used. One such recycling method is the use of polymeric waste materials as precursors for the production of activated carbons [1]. The production of carbon materials from this type of waste has been the subject of numerous studies, in which the following materials were used: poly(ethylene terephthalate) (PET) [3,4,5,6,7,8,9,10,11], polystyrene (PS) [12,13], polyethylene (PE), poly(vinyl chloride) (PVC), polyacrylonitrile (PAN), polypropylene (PP), poly(methyl methacrylate) (PMMA) [14], polycarbonate (PC) [15,16], etc.

These types of plastics are applied in our daily lives for the production of disposable tableware, fabrics, bags, coatings and wiring, structural elements, equipment, and several other materials, to name just a few [1,13]. Notably, not every polymer is known to be a good carbon precursor. However, most polymers can be applied for the production of activated carbons, and are thus effective materials [13]. An important parameter is the choice of an appropriate carbon precursor, which should be characterized with consideration of availability, cost, and, above all, a high carbon content and a low ash content. Taking into account the economic and environmental benefits, the use of waste as carbon precursors can be an attractive alternative [17].

To obtain active carbons characterized by very good properties (large specific surface area and established porosity), it is necessary to address the following points: which polymer should be chosen; what carbonization and activation conditions should be selected; what activating agent should be used; and whether additional modification is needed, and if so, with which activating agent and under what conditions [13].

During the process of activation, there are two main distinguishable procedures: physical and chemical activations. The most common method from the point of view of commerce is physical activation, because chemical reagents are not used, and thus, the costs and amounts of impurities are reduced [18,19,20]. The material carbonized previously in the partially oxidizing atmosphere (H_2_O, CO_2_, or their mixture) is gasified over a range of temperatures from 700 to 1000 °C [21,22]. Volatile substances, such as aliphatic carbons and heteroatoms, are released during the carbon precursor carbonization. Moreover, the residual carbon atoms assume the structures of aromatic sheets, which are flat and randomly cross-linked. Then, as a result of gasification during the physical activation, the irregularly arranged carbon sheets lead to the formation of free spaces, corresponding to the initial porosity, which can be further developed. It is possible to create new pores by removing material that possibly blocks the entrance to existing pores. Finally, there is a clear development of the accessible porosity and an increase in the surface area [18]. However, chemical activation takes place in the following two ways: the first includes carbon precursor impregnation using a chemical activator, which is followed by high-temperature heating (activation/carbonization) [23]; the second method comprises two stages, i.e., carbon precursor carbonization (300 to 600 °C) and then impregnation with a chemical agent and activation (700 to 1200 °C) [24]. Carbon material produced by chemical activation is more efficient than that produced by physical activation. However, it is more expensive and harmful for the environment [18,25].

A serious global problem is the degradation and pollution of the natural environment [26]. There is no doubt that municipal and industrial wastewater pollutes surface waters; colored organic compounds are undoubtedly the main group of such pollutants [27]. Today, many industries, e.g., textile, paper, dyestuffs, and plastics, use dyes to color their products. As a result, manufacturing industries generate a huge amount of colored wastewater, which adversely affects the aquatic environment, hindering the penetration of light, and thus preventing aquatic flora photosynthesis [28,29].

One of the most common dyes is methylene blue (MB, cationic dye), which is very frequently applied in industry, in the dyeing of cotton, wood, or silk [29]. Moreover, this dye is used in the cosmetic, food, and pharmaceutical industries [30]. MB was also proven to have a therapeutic effect: the neutralization of heparin and treatment of malaria (36–72 mg/kg for 3 days). Another application is the treatment of vasoplegia after transplant operations. However, MB, as a component of sewage, enters water ecosystems, posing a danger to living organisms. In humans, MB can lead to shock, increased heart rate, vomiting, tissue necrosis, cyanosis, jaundice, and other side effects. In plants, the presence of MB inhibits their growth, reduces pigments, etc. [30]. Malachite green (MG, cationic dye), due to its versatile use in aquaculture as antiparasiticin, in the leather industry, and as a coloring agent in the paper, silk, and wool industries, is also very popular [31]. MG is toxic, carcinogenic, and mutagenic. Moreover, it has adverse effects on the sensory organs and the respiratory system, and can also lead to reduced fertility. In summary, dyes that end up in aquatic environments can cause many health problems; thus, they must be removed [31,32,33].

Therefore, to protect the environment, it is necessary to explore appropriate adsorbents (cheap, but above all, effective). Among the various purification technologies, activated carbon adsorption is one of the most effective and reliable methods of physicochemical purification [28]. The adsorption effectiveness of activated carbons (ACs) is due to their large surface area as well as the significant number of oxygen functional groups [17].

Large amounts of diverse forms of waste are produced worldwide. They are characterized by different physical properties, affected by their nature, the method of storage, and the technological processes that they undergo. The adsorbents obtained from waste CDs contribute to the “second life”. The waste materials include of polycarbonates (more than 95% of the total weight of the disc). A disc surface is covered with a thin layer of silver or aluminum. Many billions of CDs are still used or became waste. Therefore, they should be got rid of by their processing not doing harm to the environment. CDs were rarely examined as carbon precursors. However, there are few literature reports on this topic [14,15,16]. This motivated us to obtain new effective adsorbents with developed porosity based on this type of precursors. However, the literature reports much more about PETs as precursors [3,4,5,6,7,8,9,10,11].

The authors believe that the presented research result fit the global trend aimed at obtaining and characterizing carbon materials from various wastes. As follows from worldwide research, the use of polymer waste for the production of activated carbons lends a fresh perspective to the protection of the natural environment and fuel management. However, a very advantageous solution deserves emphasis which justified the research subject. 

The objective of the investigations was to prepare activated carbons from polycarbonates-polymer waste (waste CDs) as a result of carbonization and then physical activation (with CO_2_) as well as to characterize the physicochemical properties taking into account the adsorption properties of the dyes, i.e., methylene blue and malachite green. The research is innovative, especially when it comes to MG adsorption because, to our knowledge, there are no literature reports on the adsorption of this dye on activated carbons obtained from polymeric materials. The presented investigations were based on carbon adsorbents obtained by physical activation due to being cheaper and eco-friendly (compared to chemical activation), resulting in preparation of effective carbon adsorbents.

## 2. Materials and Methods

### 2.1. Materials and Reagents

Waste CDs were used as a carbon precursor. The organic dyes methylene blue hydrate, purity 96% (C_16_H_18_ClN_3_S *H_2_O, M_mol_ = 319.85 g mol^−1^), and malachite green carbinol hydrochloride, purity 85% (C_23_H_26_N_2_O *HCl, M_mol_ = 382.93 g mol^−1^), used as model adsorbates, were purchased from Sigma-Aldrich (Schnelldorf, Germany).

### 2.2. Preparation of Adsorbents

At first, the polymer waste in the form of CDs was cut into small pieces. Then, the prepared material was poured with 10 wt% hydrochloric acid solution (Chempur, Piekary Śląskie, Poland) and left for 12 h. After washing with distilled water, it was dried overnight. Thus, the CDs top layer was removed. Using the instructions in [14], slightly modified, materials were obtained. Placed in a quartz boat, they were carbonized in a nitrogen atmosphere (gas purity 5.0, 20 dm^3^ h^−1^) at 500 °C. The heating rate was 1 °C min^−1^. The process of annealing at this temperature lasted 1 h. Physical activation using CO_2_ was applied to develop the porous structure. At first, carbon was heated at room temperature to 940 °C in a nitrogen atmosphere (gas purity 5.0, 20 dm^3^ h^−1^), then the gas (N_2_) was changed to CO_2_ to carry out the activation process. Activation took place at 940 °C at CO_2_ atmosphere (99.998%) for 4 h, 6 h, and 8 h (the CO_2_ flow rate: 5 dm^3^ h^−1^). Next, CO_2_ was changed again to N_2_ (20 dm^3^ h^−1^) to cool the system to room temperature. The flow of N_2_ and CO_2_ during carbonization and activation was verified by means of a rotameter. The prepared carbons were: C-CD (4 h), C-CD (6 h), and C-CD (8 h). From 1 g of carbon precursor, 0.35 g, 0.31 g and 0.27 g of each of the mentioned carbons were obtained, respectively. After pyrolysis, the carbons were crushed and sieved. A fraction of 0.4 to 0.8 mm was selected for this study.

**Figure 1:**
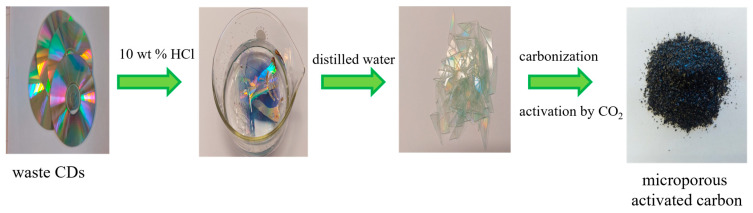
Scheme of the microporous activated carbons preparation.

The scheme of microporous activated carbons preparation is presented in Figure 1.

### 2.3. Carbon Materials Characterization

Low-temperature nitrogen adsorption/desorption isotherms (−196 K) were used for determination of carbon materials porous structure by means of the volumetric adsorption analyzer ASAP 2020 (Micromeritics, Norcross, GA, USA) (Structural Research Laboratory of Jan Kochanowski University in Kielce). Before making the adsorptive measurements, all carbon samples were degassed at 200 °C for 2 h. The porous structure standard parameters, pore volume, specific surface area and pore size distribution, were determined using the nitrogen adsorption isotherms. The specific surface area (S_BET_) was determined in the relative pressure range from 0.05 to 0.20 considering the single nitrogen molecule surface (0.162 nm^2^) [34]. The total pore volume (V_t_) was determined from the point of the adsorption isotherm which corresponded to the relative pressure p/p_0_ = 0.99 [35]. The non-local density functional theory (NLDFT) was applied for calculation of pore size distribution (PSDs) functions for the slit-shaped pores of carbon with surface energetic heterogeneity as well as geometrical corrugation [36,37] by means of the numerical program SAIEUS (Micromeritics).

The morphology of carbon materials was studied by means of SEM Zeiss mod. Ultra Plus, EDS Bruker Quantax 400 (Bruker, Karlsruhe, Germany). During the measurements, the voltage was 2 kV. Energy-dispersive X-ray spectroscopy (SEM/EDX, acceleration: 15 kV) was used in quantitative analyses.

The elemental analysis (CHNS) was performed using the Elementar Vario Micro Cube analyzer (Elementar, Langenselbold, Germany). Before the measurements, the samples were dried to constant weight.

The degree of carbon skeleton structure ordering was determined recording the Raman spectra using a spectrometer (Raman Station 400 F, Perkin Elmer, Waltham, MA, USA) with the thermoelectrically cooled CCD detector and diode laser. The wavelength measurement was 785 nm and the power was 350 mW. Each sample was dried earlier. Five scans were made for each sample at the scanning time 20 s. The resolution was 1 cm^−1^.

X-ray diffraction analysis was performed on the PAN-alytical X’Pert PRO MPD X-ray diffraction system (PANalytical Inc., Westborough, MA, USA) using Cu Kα radiation (40 kV, 40 mA, step 0.02°, range 10° ≤ 2θ ≤ 50°). The crystal structure was analyzed using the PDF-2+ 2009 Database.

Derivatograph C (Paulik, Paulik and Erdey, MOM, Budapest, Hungary) was applied for determination of thermal stability as well as volatile and fixed carbon share in the samples. An amount of 10 mg of materials was put in a corundum crucible and Al_2_O_3_ was used as the reference material. The range of temperatures was from 20 to 1000 °C in either the air or inert (N_2_) atmosphere with the 10 °C/min heating rate. The TG, DTG, and DTA curves were registered.

The content of volatile carbon, which is a less humified organic matter (%VC) was estimated based on the data TGA in the N_2_ atmosphere in the temperature range from 150 to 900 °C, assuming the moisture desorption temperature to be up to 150 °C. Complete material thermooxidation in an O_2_ atmosphere at up to 1200 °C results in ash (%A) as an inorganic residue. The amount of fixed carbon (% FC), being the more humified organic matter, was calculated as the difference of TG%_1200,O2_, and TG%_900,N2_ [38]. The share of thermostable fraction (C_thermo_) being poorly thermodegradable was determined as stable matter (%FC) per the sum of volatile (%VC) and fixed substances (% FC) [39]. Using this parameter, the stability of organic matter in the carbonaceous materials can be estimated [40].

The infrared spectra were recorded by means of a Perkin-Elmer Spectrum 400 FT-IR/FT-NIR spectrometer (Perkin-Elmer, Waltham, MA, USA) with a smart endurance single-bounce diamond, attenuated total reflection (ATR) cell. The 4000–650 cm^−1^ spectra were recorded due to the co-addition of 500 scans obtained at the 4 cm^−1^ resolution. The samples were subjected to drying and powdering in the agate mortar before the measurements.

The surface oxygen acidic and basic functional groups were determined by means of Boehm’s titration method [38,41,42]. An amount of 0.2 g carbon was dispersed in the sodium bicarbonate, sodium carbonate, sodium hydroxide and sodium ethoxide solutions for functional acidic groups determination. The total basic groups were determined using hydrochloric acid. Solution shaking at room temperature lasted 48 h. Then, the samples were filtered and 10 cm^3^ of filtrate was titrated using 0.1 mol dm^−3^ HCl for determination of acidic groups and 0.05 mol dm^−3^ NaOH for determination of the total basic groups.

The point of zero charge (pH_pzc_) was analyzed by means of the method presented in [43,44,45]. The 0.01 mol dm^–3^ NaCl solutions were prepared and then the pH was brought to 3–12 by adding 0.1 or 1 mol dm^–3^ HCl and 0.1 or 1 mol dm^–3^ NaOH. Approximately 0.15 g of the materials were put into the 100 cm^3^ flasks. Then, they were poured over using 50 cm^3^ sodium chloride solutions of different pH values. Shaking in an incubator (Orbital Shaker—Inkubator ES-20, Grant-bio, Wasserburg, Germany) at 25 °C constant temperature and 200 rpm speed lasted 4 h. Next, the final pH was measured using a pH-meter (inoLab pH 730, WTW GmbH, Weilheim, Germany). In the next step, the final pH and the initial pH relationships were determined. The pH_pzc_ is defined as the point of this line with the pH_initial_ = pH_final_ one intersection [43,44].

### 2.4. Adsorption Studies

Adsorption experiments were performed in 100 mL Erlenmeyer flasks in the incubator for a definite time. Adsorption kinetics: the concentration of MB and MG was 300 mg dm^−3^, with the time from 15 to 600 min for MB and 15 to 360 min for MG. Adsorption isotherms: the concentrations of MB and MG were 200 300, 400, 600, 800, 1000, 1200 mg dm^−3^; experimental time: equilibrium conditions—480 min for MB, and 300 min for MG. The carbons were weighed to be 0.1 g, then poured with 50 cm^3^ MB and MG solutions.

The spectrophotometric method (SP-830 Plus from Metertech, Poznań, Poland) was applied at the 465 nm (MB) and 615 nm (MG) wavelengths for the dye concentrations determination before and after the adsorption process. The temperature effect on the adsorption process was studied. The test temperatures were 298 K, 308 K and 315 K and the stirring rate was 200 rpm. All adsorption studies were carried out in three series, and the results were averaged.

The calculation of the amount of dyes being adsorbed at equilibrium (*q_e_*, adsorption capacity, mg g^−1^) was based on Equation (1):

(1) $$q_{e}=\frac{(C_{0}-C_{e})V}{m}$$

where *C*_0_ and *C_e_* are the initial and equilibrium concentrations of dye solutions (mg dm^3^), *V* is the volume of the dye solution (dm^3^), and *m* is the mass of carbon materials (g).

The adsorption kinetics of MB and MG from aqueous solutions on the carbon materials was described using the pseudo-first-order model, also known as the Lagergren equation (Equation (2)) [46], the pseudo-second-order model also called the Ho equation (Equation (3)) [47] as well as the Weber–Morris intraparticle diffusion model (Equation (4)) [48].

(2) $$\ln\left(q_{e}-q_{t}\right)=\ln q_{e}-k_{1}t$$

(3) $$\frac{t}{q_{t}}=\frac{1}{k_{2}q_{e}^{2}}+\frac{t}{q_{e}}$$

(4) $$q_{t}=k_{id\;}t^{1/2}+c$$

where *k*_1_—the pseudo-first order rate constant (min^−1^); *k*_2_—the pseudo-second order rate constant (g mg^−1^ min^−1^); *t*—the time of contact between the adsorbent and the adsorbate (min); *q_e_*—the adsorption value after the equilibrium stabilization (mg g^−1^); *q_t_*—the adsorption value at a given time *t* (mg g^−1^); *k_id_*—the intraparticle diffusion rate constant (mg g^−1^ min^−1/2^); and *c*—the intercept, which represents the thickness of the boundary layer (mg g^−1^).

Two equilibrium isotherm models, namely Langmuir [49] (Equation (5)) and Freundlich [50,51] (Equation (6)), were used.

(5) $$\frac{C_{e}}{q_{e}}=\frac{1}{q_{m}}C_{e}+\frac{1}{q_{m}\;K_{L}\;}$$

(6) $$logq_{e}={\log K}_{F}+\frac{1}{n}C_{e}$$

where *q_m_*—the maximum adsorption capacity corresponding to the total monolayer coverage on the adsorbent surface (mg g**^−^**^1^); *K_L_*—the Langmuir constant (dm^3^ g**^−^**^1^), *K_F_*—the Freundlich isotherm constant (mg^(1−1/*n*)^ (dm^3^)^1/*n*^ g**^−^**^1^); *n*—the empirical constant describing the heterogeneity of the adsorbent surface.

Free enthalpy (Δ*G*) was determined to approximate the nature of the adsorption processes of the tested systems (Equation (7)) [52].

∆*G* = −*RT*ln*K_L_* (7)

## 3. Results

### 3.1. Characterization of Carbon Materials

#### 3.1.1. Porous Structure of Carbon Materials

**Figure 2:**
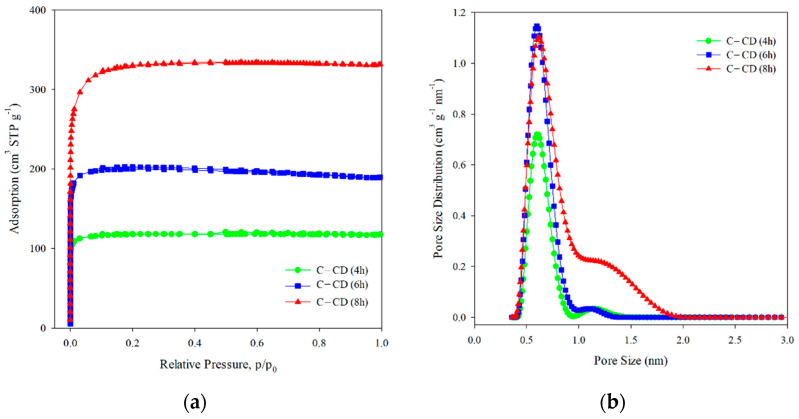
Nitrogen adsorption/desorption isotherms (**a**) and pore size distribution curves (**b**) for the studied activated carbons.

Based on the IUPAC classification, the experimental adsorption–desorption N_2_ isotherms of activated carbons (Figure 2a) are of type I [53,54], indicating their microporous character. The structural parameters calculated based on the adsorption isotherms are given in Table 1. As follows from the pore size distribution curves (Figure 2b), there is one complex peak in the micropore range, indicating the bimodal micropores structure. The DFT method was applied for determination of the micropores dimension (Table 1). According to the IUPAC classification [55], they are ultramicro- (0.4 < d < 0.7 nm) and supermicropores (0.7 < d < 2 nm). The BET surface was the largest for C-CD (8 h) sample (1136 m^2^ g^−1^) compared to the other carbons. This tendency is also observed in the case of other parameters, i.e., the total pore volume (0.51 cm^3^ g^−1^), and the micropore volume (0.49 cm^3^ g^−1^) for C-CD (8 h). In the case of shorter activation time, i.e., 4 h and 6 h, the obtained adsorbents had less developed porosity and thus smaller S_BET_ values (4 h: 399 m^2^ g^−1^, 6 h: 679 m^2^ g^−1^), V_t_ (4 h: 0.18 cm^3^ g^−1^, 6 h: 0.29 cm^3^ g^−1^), and V_micro_ (4 h: 0.18 cm^3^ g^−1^, 6 h: 0.29 cm^3^ g^−1^). If the values of the specific surface area are taken into account, then for 6 h of activation, a 2-fold surface area was obtained, while for 8 h, its value was 3-fold higher compared to the material after 4 h of activation. To sum up, the parameters of the porous structure increase with the increasing time of CO_2_ activation. The analysis of the micropore dimensions showed a similar size for all carbons (ultramicropores: 0.60–0.61 nm and supermicropores: 1.14–1.26 nm Table 1).

#### 3.1.2. SEM/EDS Analysis

**Figure 3:**
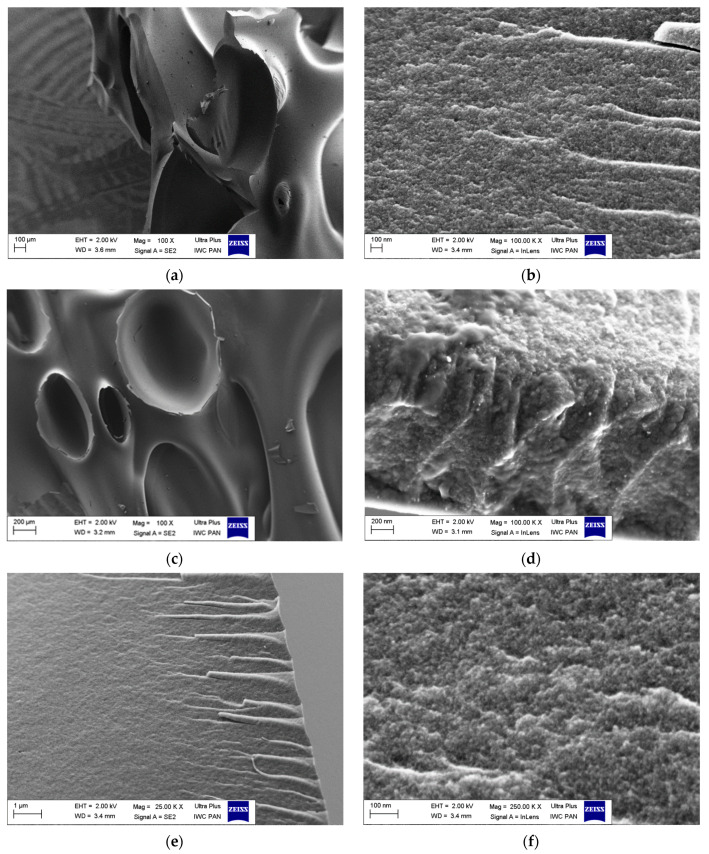
SEM images of microporous carbons: (**a**,**b**) C-CD (4 h); (**c**,**d**) C-CD (6 h); (**e**,**f**) C-CD (8 h).

Figure 3 presents the activated carbon morphologies observed by means of SEM. The carbons prepared from the polymer waste possess a layered structure, indicating their arrangement. Figure 3b,d,f shows that activation time extension results in a less smooth and rougher surface, indicating greater development of porosity. Such conclusions are consistent with the results of BET studies, which show that activation causes an increase in the specific surface area and porosity (Table 1).

**Figure 4:**
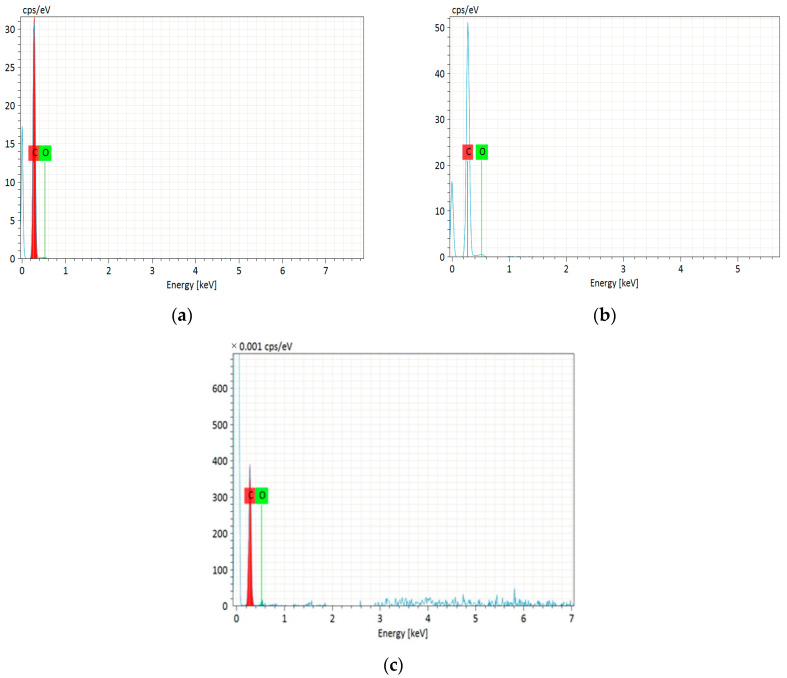
EDS spectra of the obtained carbons: (**a**) C-CD (4 h); (**b**) C-CD (6 h); (**c**) C-CD (8 h).

The EDS analysis (Figure 4a–c) confirmed the presence of carbon and oxygen in the synthesized activated carbons. The detailed data are presented in Table 2. The tested adsorbents contain 94.46 to 97.89% *w*/*w* carbon, and 5.54 to 2.11% *w*/*w* oxygen. C-CD (4 h) contains the most carbon, C-CD (6 h) slightly less, and C-CD (8 h) the least. Simultaneously, the oxygen content gradually increased, indicating that the surface functionalities formation in the activation process is observed. To sum up, it can be stated that the porous structure is more developed with the smaller carbon content.

#### 3.1.3. CHNS Analysis

The elemental composition of the obtained carbon materials is presented in Table 2. The results proved that the activation time has a significant impact on the carbon content. As the activation time increases, the carbon percentage decreases from 99.30% to 96.19%. This trend is consistent with the results of the EDS analysis (Table 2). The CHNS analysis did not show nitrogen or sulfur. Therefore, it can be assumed that oxygen, the content of which increases with the increasing activation time, is the residue. The results obtained by EDS and CHNS are slightly different since EDS is a surface analysis while CHNS allows for the analysis of the entire sample volume.

#### 3.1.4. Raman Analysis

**Figure 5:**
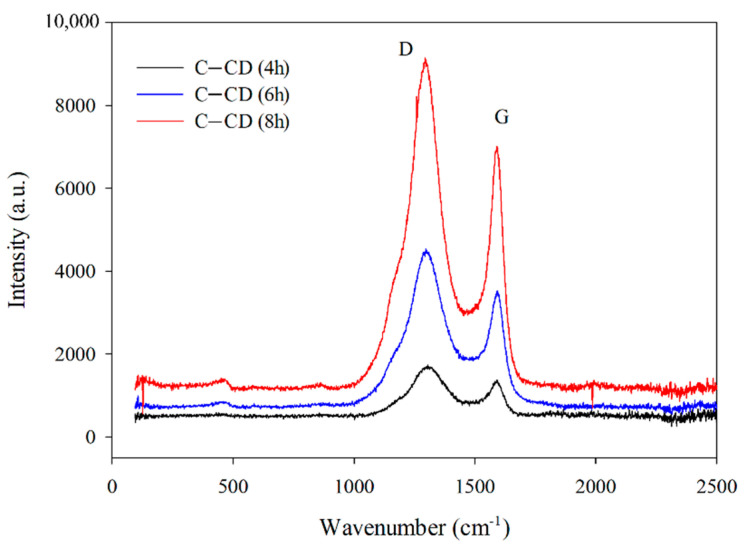
Raman spectra for the carbons.

The activation time has a significant impact on the graphitization degree of activated carbons which was proved by the Raman spectroscopy (Figure 5). Two broad overlapped bands with the maxima at 1360 cm^−1^ and 1590 cm^−1^ are characteristic of carbon materials and correspond to the D and G bands. The G-band is typical of monocrystalline graphite originating from the tensile vibrations of sp^2^-hybridized carbon bond pairs in the rings of graphene layers and the structure of graphitized carbon. Well-graphitized carbon is characterized by a narrow and high band. The band widening indicates defects in the graphene planes and the sp^2^ carbon structure. With the increasing disorder, the D-band appears, indicating the amorphous nature and existence of defects in the sp^3^ hybridization graphite structure. Determination of the correlation between the G and D bands position and their intensity (I_D_/I_G_) allows to describe the structural properties of carbon materials. An increase in the I_D_/I_G_ peak intensity ratio indicates a decrease in the carbon graphitization degree [56].

Figure 5 shows the Raman spectra recorded for the activated carbons. An increase in the D and G bands intensity with the increasing activation time is noticeable (Figure 5). The bands are wide, indicating the existence of structural defects. The D-band to G-band intensity ratio (I_D_/I_G_) is considered a determinant of the amorphous phase in carbon materials. The I_D_/I_G_ ratios (Table 2) increase from 1.24 to 1.30 with the increasing activation time, indicating an increase in the graphitization degree. The obtained values point out to the increasing share of disordered graphene structures containing carbon atoms with sp^3^ hybridization (decrease in the graphitization degree).

#### 3.1.5. XRD Analysis

**Figure 6:**
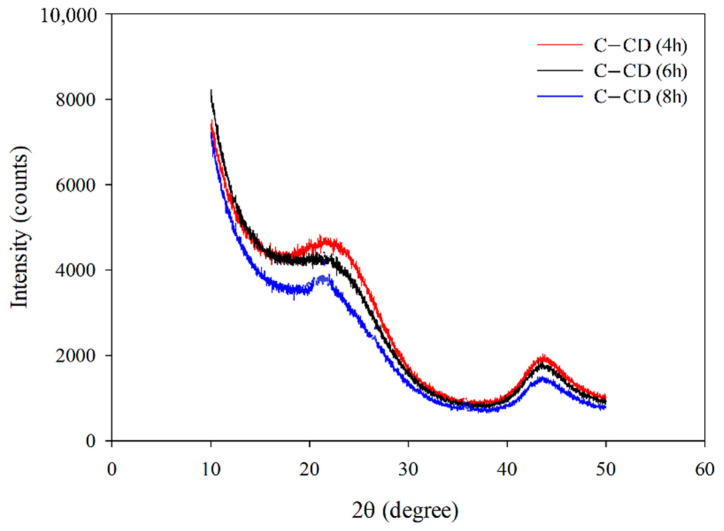
XRD patterns for the studied carbons.

Figure 6 presents the XRD spectra of activated carbons. Wide, bloated peaks, reaching the maximum at 2θ = 23° characteristic of the non-graphitic structures of carbons can be observed. This indicates that the materials possess the amorphous structure. Very low and wide diffraction peaks can be observed at 2θ = 43° which indicates the presence of a small number of graphite structures [57].

#### 3.1.6. Thermal Analysis

**Figure 7:**
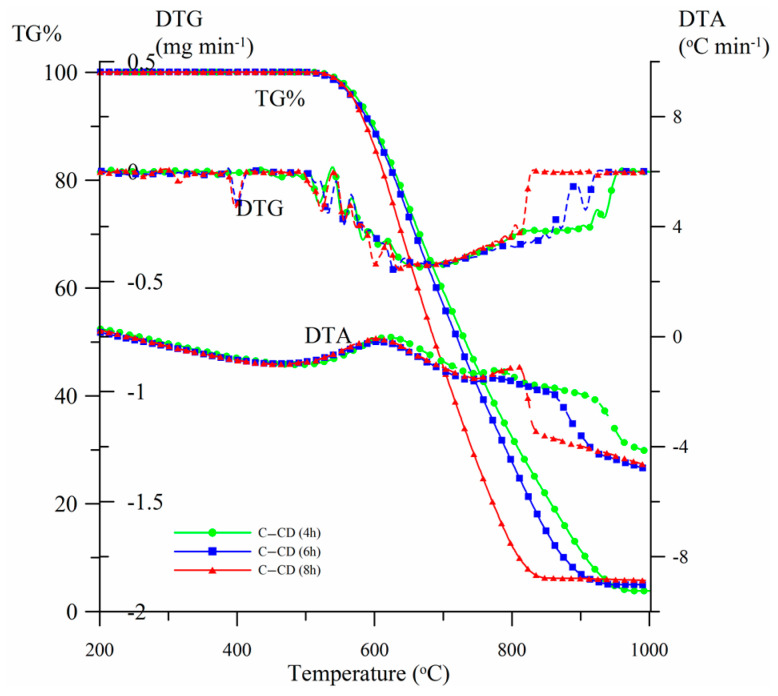
Activated carbons TG%, DTG and DTA curves.

The thermal stability of the materials was estimated by means of thermal analysis. Figure 7 presents the TG%, DTG and DTA curves for the carbons obtained from CDs activated for 4, 6 and 8 h (samples C-CD (4 h), C-CD (6 h) and C-CD (8 h), respectively). As follows from the mass loss curves (TG%) analysis, the thermal stability of all materials proved to be up to ~ 530 °C. The carbon material is completely thermally degraded over the range of temperatures, 850–975 °C, taking the activation time into account. The longer the activation time, the faster the oxidative degradation of carbon is (DTG range, Table 3). These changes are reflected in the DTG and DTA curves course. The DTG curves show a broad minimum in the mass loss temperature range. The uniform course of the curves indicates carbon’s structural homogeneity. Developed maxima are observed on the DTA curves at ~600 °C. The curves course curves and the analysis of the ash content (%A) indicate that oxidation in the CO_2_ atmosphere causes partial degradation of organic compounds (Table 3). The carbon ash content increases from 3.7% (C-CD (4 h)) to 5.8% (C-CD (8 h)) when the activation time is extended. The carbon structure in all materials is similar; however, a slight decrease in the content of volatile organic compounds (%VC) is observed, with a simultaneous increase in the value of fixed carbon (%FC) and the thermal stability index (C_thermo_, Table 3).

#### 3.1.7. ATR-FTIR Analysis

**Figure 8:**
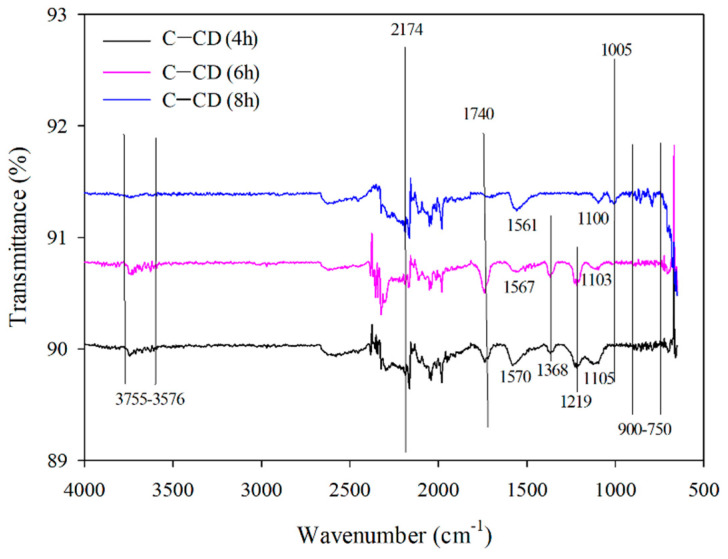
FTIR spectra of the carbons.

ATR-FTIR spectra of all activated carbons are presented in Figure 8. The bands in the 3755–3576 cm^–1^ range correspond to the asymmetrical and symmetrical tensile vibrations of -OH groups for all carbons [58,59,60,61]. Moreover, the 2174 cm^–1^ band results from the presence of CO in the carbons [59]. However, the bands at 1740 cm ^–1^ with larger intensities for C-CD (6 h) and C-CD (8 h) and a very low intensity for C-CD (4 h) correspond to the vibrations due to the double bonds presence between the atoms of carbon or those of carbon and oxygen [62,63]. The bands at 1570 cm^–1^ for C-CD (4 h), 1567 cm^–1^ for C-CD (6 h) and 1561 cm^–1^ for C-CD (8 h) are characteristic of the C=C bonds [64]. The bands of the differentiated intensity in the 1500–1100 cm^–1^ range are due to the carbonyl groups C=O [65,66] (1368 cm^–1^, 1219 cm^–1^ and 1105 cm^–1^ for C-CD (4 h); 1368 cm^–1^, 1103 cm^–1^ for C-CD (6 h); 1100 cm^–1^ for C-CD (8 h) However, the presence of these groups is not confirmed by the Boehm method. The band of 1005 cm^–1^ is characteristic of the C-OH tensile vibrations only in the case of C-CD (8 h) sample [66] The bands in the range from 900 to 750 cm^−1^ indicate the bending vibrations of C-H (slightly larger intensity for C-CD (8 h) and smaller intensity for the other two carbons) [58].

#### 3.1.8. pH_pzc_

**Figure 9:**
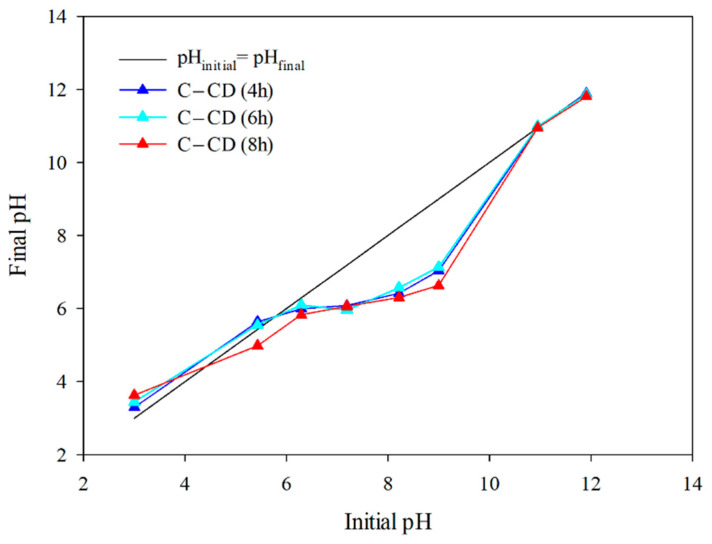
pH_pzc_ for the carbons (pH drift method).

The surface charge (pH_pzc_) value of the carbons is largely affected by the functional groups present on their surface. The pH value, at which the net surface charge of the adsorbent is zero, can be defined as pH_pzc_. In a solution with pH < pH_pzc_ the charge of carbon surface is positive, but in the case of pH > pH_pzc_, it is negative. The relationship of pH_final_ as a function of pH_initial_ is presented in Figure 9. The values of pH_pzc_ determined from the graph (Figure 9) are: for C-CD (4 h): 5.77; for C-CD (6 h): 5.70; and C-CD (8 h): 4.40. The charge of the carbons is negative. It is essential that the adsorption properties of the MB and MG adsorbates are determined by the surface charge. Taking into account the electrostatic adsorbent-adsorbate interactions, the cationic MB and MG adsorption should be intensive on the surface of carbon being negatively charged at pH > pH_pzc_.

#### 3.1.9. Boehm Method

Table 4 presents the oxygen functional groups content on the surface of the activated carbons. Both types of functional groups, acidic and basic ones, are present on the adsorbents surface. In the case of C-CD (4 h), the basic groups (0.312 mmol g^−1^) dominate over the acidic ones (0.249 mol g^−1^); for C-CD (6 h), it is analogous (0.359 mmol g^−1^ and 0.186 mmol g^−1^, respectively); for C-CD (8 h), the acidic groups (0.412 mmol g^−1^) prevail over the basic ones (0.312 mmol g^−1^). In the case of the tested adsorbents, only the presence of phenolic groups was confirmed. The remaining groups could not be determined using the Boehm method. The carbonyl groups, not detected by the Boehm method, was confirmed by the FTIR analysis. This can be due to their small amount on the carbon materials surface. Moreover, the 8 h activation gave a large increase in the surface functional groups amount as proved by the FTIR analysis (Figure 8), the EDS microanalysis (Table 2) and the Boehm method (Table 4).

### 3.2. Adsorption Study

#### 3.2.1. Adsorption Kinetics

**Figure 10:**
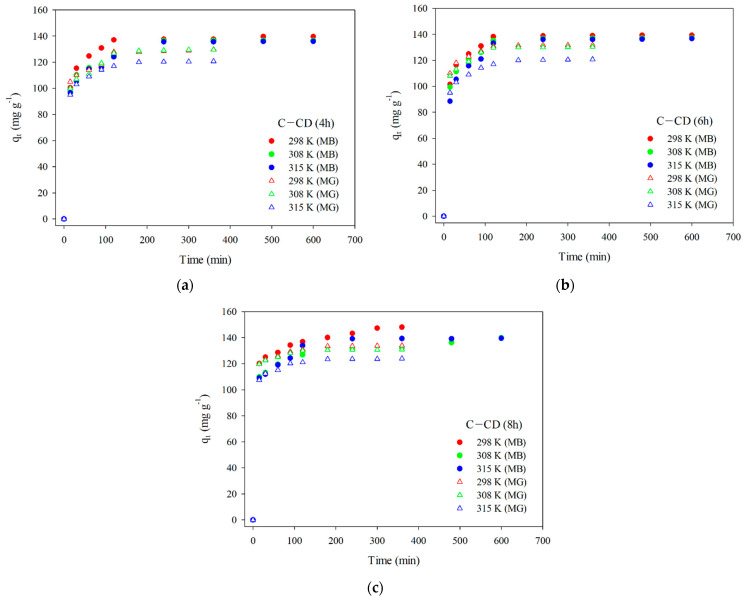
Experimental kinetic data (MB and MG) for C-CD (4 h) (**a**), C-CD (6 h) (**b**), and C-CD (8 h) (**c**).

The MB and MG adsorption on the adsorbents was described using the pseudo-first-order kinetic (Equation (2)) [46], pseudo-second-order kinetic (Equation (3)) [47], and intraparticle diffusion (Equation (4)) models [48] (Table 5). The pseudo-first-order model characterizes physical adsorption, in which the physical interactions between the adsorbent and the adsorbate are the adsorption limiting. The pseudo-second-order kinetic model characterizes chemisorption, which is also adsorption limiting step. The Weber–Morris intraparticle diffusion model is used to elucidate the adsorption mechanism which proceeds in three stages [67,68,69]: (1) diffusion of adsorbate molecules into the outer surface of the adsorbent and adsorption on its surface; (2) intraparticle diffusion and gradual adsorption of adsorbate; (3) interactions between the adsorbate molecules and the adsorbent active sites in the equilibrium stage. The experimental kinetic data for MB and MG are presented in Figure 10a–c. The equilibrium of the MB adsorption process was established after 480 min, and in the case of MG after 300 min for all materials.

**Figure 11:**
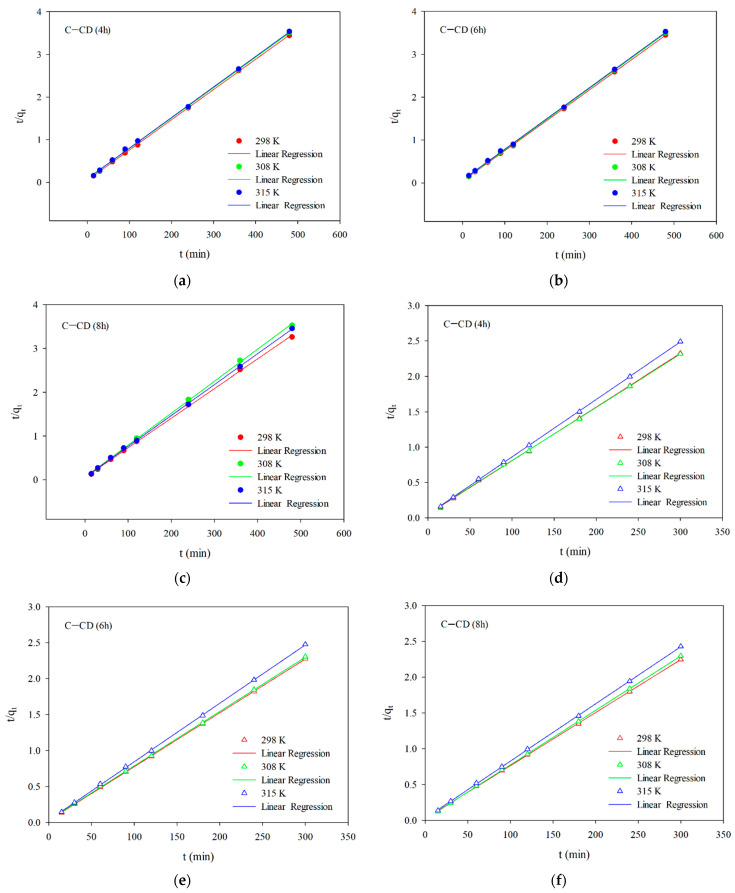
Linear relationships for the pseudo-second-order model (**a**–**c**) for MB and (**d**–**f**) for MG.

Figure 11a–f presents the linear relationships for the pseudo-second-order (Equation (3)) model of adsorption of MO (Figure 11a–c) and MB (Figure 11d–f) on the adsorbents. Table 5 and Table 6 show the kinetic parameters and the correlation coefficients R^2^. Based on the obtained results (Figure 11a–f, Table 5 and Table 6) and taking into account the value of the R^2^ coefficient for MB (0.9995–0.9999) and for MG (0.9995–1.0000) one can state that the adsorption process speed can be described by the pseudo-second-order model. As fitting to this model show the dyes adsorption process depends largely on the active centers accessibility [70,71]. According to the pseudo-second-order kinetic model adsorption of MB and MG indicates chemisorption (the chemical interactions between adsorbate molecules and the adsorbent surface) [68]. This confirms permanent binding of the adsorbate molecules to the adsorbent surface.

**Figure 12:**
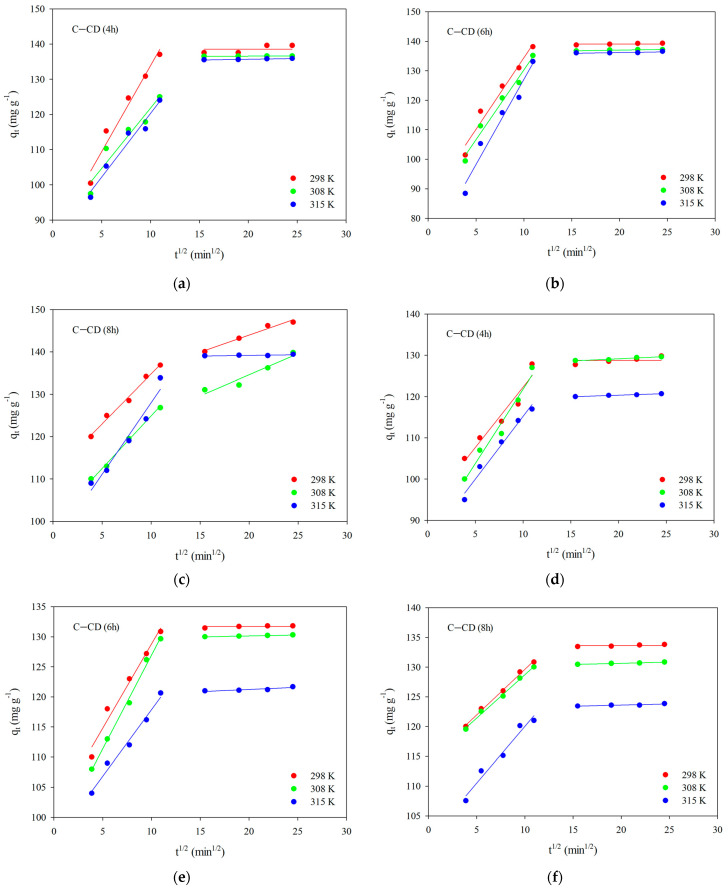
Intraparticle diffusion of MB (**a**–**c**) and MG (**d**–**f**) for C-CD (4 h) (**a**,**d**), C-CD (6 h) (**b**,**e**) and C-CD (8 h) (**c**,**f**).

The Weber–Morris intraparticle diffusion model (Equation (4)) was applied to obtain better information about the adsorption mechanism. The relationships in Figure 12 will promote explanation how diffusion effects on the dyes adsorption. Two linear sections were obtained for the systems, indicating that the adsorption rate is influenced not only by intraparticle diffusion but also by other processes [72]. The first of the linear sections deals with intraparticle diffusion, which can be interpreted as diffusion in small pores (micropores), whereas the second one presents the adsorption equilibrium. In the case of the first section, the diffusion constant values *k_id_* were calculated (Table 7 and Table 8). On the other hand, the determined “*c*” values (Table 7 and Table 8) are non-zero and they are positive, indicating that intraparticle diffusion is not the adsorption process limiting step for the systems being studied.

In the case of MG adsorption, the equilibrium was established much faster than in the case of MB, which can be also evidenced by the values of *k_id_*, which are lower for MG (Table 8).

#### 3.2.2. Adsorption Isotherms

**Figure 13:**
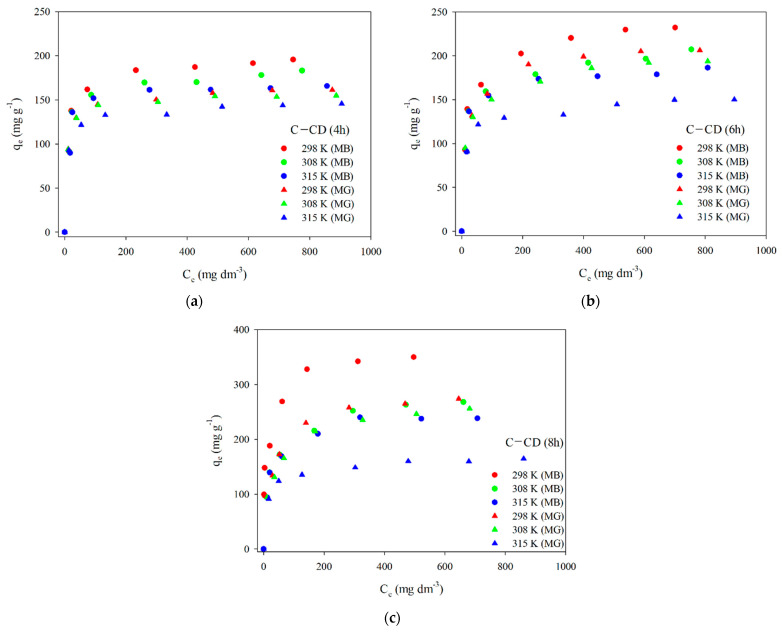
Experimental adsorption isotherms of MB and MG for C-CD (4 h) (**a**), C-CD (6 h) (**b**) and C-CD (8 h) (**c**).

To present the adsorbed dyes’ molecules distribution on the surface and interactions with adsorbents, it is essential to determine the adsorption isotherm model. Figure 13 presents the experimental adsorption isotherms of MB and MG on the carbons. Taking into account the experimental and calculated *q_e_* values, adsorption decreases with the increasing temperature, indicating the exothermic nature of the process. The experimental data were fitted to the linear forms of two models: Langmuir (Equation (5)) and Freundlich (Equation (6)). The Langmuir isotherm is used to describe the monolayer adsorption on a homogeneous surface on which one molecule of the adsorbate occupies one active site of the adsorbent. According to the Langmuir model adsorption depends on the temperature, concentration and magnitude of adsorption energy. In this case multilayer adsorption is not possible, the adsorption energy is constant. Due to the energetically homogeneous surface and the lack of interactions of adsorbate molecules with each other (there are only interactions of adsorbate molecules with the adsorbent surface), the strength of intermolecular attraction decreases with distance, all active sites of the adsorbent are identical and energetically equivalent. Moreover, the adsorbent has a finite capacity—the quantity of adsorbate molecules is limited [69,73,74,75].

The Freundlich isotherm describes the multilayer adsorption on the heterogeneous surfaces, often on the microporous adsorbents. It is often used to describe adsorption from aqueous solutions on activated carbons. The constant *K* describes adsorption strength and is proportional to the adsorption capacity. The higher its value, the stronger the adsorption. The *n* parameter characterizes the energetic heterogeneity of the adsorbent surface and determines the isotherm shape. For *n* = 1, the isotherm is linear, and the further the value of *n* moves away from 1, the less linear it is. A value of *n* < 1 indicates relatively high adsorption in the small concentration range. A value of 1/*n* indicates the adsorption strength. The smaller this value is, the stronger the adsorption [69,73,74,75].

**Figure 14:**
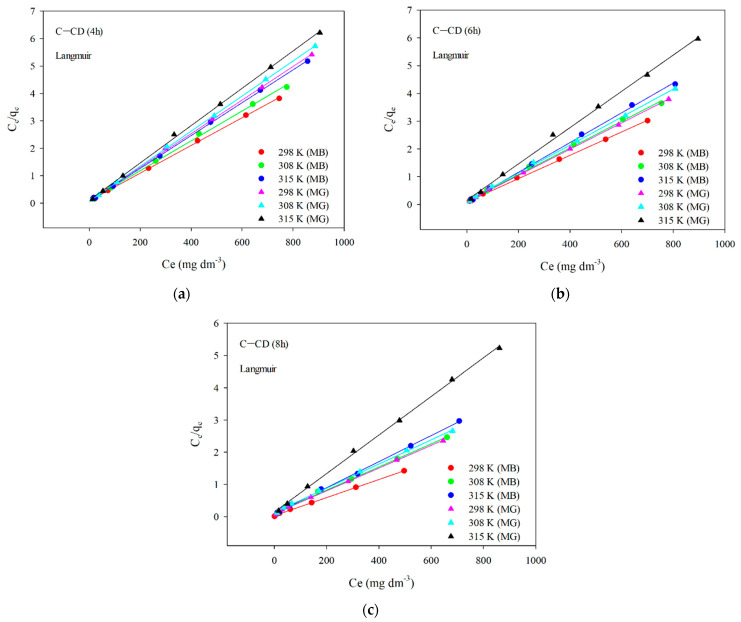
Linear relationships for the Langmuir model of MB and MG adsorption on C-CD (4 h) (**a**), C-CD (6 h) (**b**) and C-CD (8 h) (**c**).

The results of fitting to the Langmuir model are presented in Figure 14. The parameters of the equations and the correlation coefficients are summarized in Table 9 and Table 10. Analyzing the data (Figure 14, Table 9 and Table 10) and taking into account the *R*^2^ correlation coefficients large values (0.998–0.999) in the case of the Langmuir model, one can state that this is the model describing the MB and MG adsorption best on the carbon materials. The values *q_m_* are very similar to those obtained experimentally *q_e,exp_* (Table 9 and Table 10). As for the Freundlich model, the values of *R*^2^ are slightly smaller compared to those using the Langmuir model. The coefficient *K_F_* (affinity constant) shows the strength of adsorbate and adsorbent interactions. The *K_F_* values for all adsorbents were large (Table 9 and Table 10). However, the n values range from 4 to 8.7, indicating great chemical adsorption contribution, as when the *n* value is higher, the dye molecule and carbon surface interactions are stronger [76]. The value of parameter *n* is a non-integer, which indicates that the mechanism of adsorption on various adsorption centers on the adsorbent surface. The parameter *n* is greater than 1 for all materials, which means that more than one adsorbate molecule can occupy one adsorption center.

Table 11 presents the free enthalpy (Δ*G*) values (Equation (7)) for MB and MG. MB and MG adsorption on the activated carbons is spontaneous as follows from the negative ∆*G* values.

In Table 12, the results of adsorption studies (maximum capacities, *q_m_*) for MB and MG were compared with those of other similar studies to highlight the advantages of our investigations. The adsorbents obtained by us are characterized by large adsorption capacity. It is worth noting that malachite green has not been tested for adsorption from water on carbon materials obtained from polymer waste as precursors. The adsorbents were also compared with commercial carbons and it proved that the materials prepared by us exhibit similar or more effective adsorption properties. Therefore, the obtained adsorbents can compete with the commercial activated carbons.

## 4. Conclusions

Microporous activated carbons were prepared due to waste CD pyrolysis followed by physical activation with carbon dioxide. The materials’ physicochemical properties were investigated. The waste CDs proved to be an effective precursor of carbon adsorbents with developed porosity. Such materials can be successfully used in the future for adsorption of not only dyes, but also of herbicides, pharmaceuticals, trace metal ions from water, and for the storage or capture of toxic gases. Attempts to use activated carbons as supercapacitors, or to build battery electrodes, in photocatalytic studies as well as to store energy can be also of great interest.

It was proved that 8 h activation was the most effective (S_BET_ was the highest for C-CD (8 h) (1136 m^2^ g^−1^)). The obtained carbons contain more than 86% of fixed carbon and are characterized by a high coefficient of thermostability C_thermo_. The MB and MG adsorption from the aqueous solution on the carbon adsorbents was investigated. The efficiency of MB removal was significantly larger compared to that of MG for all carbons. The pseudo-second-order kinetic model can be used for description of kinetics of dye adsorption on the carbons. Adsorption is better described by the Langmuir model. Adsorption proved to be spontaneous and exothermic. In conclusion, activated carbons obtained from waste CDs as carbon precursors are effective and low-cost MB and MG adsorbents. Therefore, it can be expected that they will also adsorb other toxic and environmentally harmful dyes used on a large scale effectively.

## Figures and Tables

**Table 1:** Structural parameters of the studied carbons.

Carbon	S_BET_ (m^2^ g^−1^)	V_t_ (cm^3^ g^−1^)	V_ultra_^DFT^ (cm^3^ g^−1^)	V_micro_^DFT^ (cm^3^ g^−1^)	w_mi_^DFT^ (nm)
C-CD (4 h)	399	0.18	0.13	0.18	0.60; 1.14
C-CD (6 h)	679	0.29	0.22	0.29	0.60; 1.15
C-CD (8 h)	1136	0.51	0.22	0.49	0.61; 1.26

S_BET_—the specific surface area, V_t_—the total (single-point) pore volume obtained from the amount adsorbed at p/p_0_ ≈ 0.99, V_ultra_^DFT^—the ultramicropores volume (pores < 0.7 nm) obtained on the basis of DFT PSD, V_micro_^DFT^—the micropores volume (pores < 2 nm) obtained on the basis of DFT PSD, and w_mi_^DFT^ micropore diameter at the maximum of the PSD curve obtained by the DFT method.

**Table 2:** Results of EDS chemical composition microanalysis, CHNS analysis and the I_D_/I_G_ values.

Carbon	C (% *w*/*w*)	O (% *w*/*w*)	C (%)	H(%)	Residue O (%)	I_D_/I_G_
C-CD (4 h)	97.89	2.11	99.30	0.42	0.28	1.24
C-CD (6 h)	95.90	4.10	98.16	0.53	1.31	1.28
C-CD (8 h)	94.46	5.54	96.19	0.39	3.43	1.30

I_D_/I_G_—the graphitization degree.

**Table 3:** Activated carbons proximate analysis and thermostability indices.

Carbon	DTG Range, °C	%A	%VC	%FC	C_thermo_
C-CD (4 h)	530–850	3.7	9.7	86.6	0.899
C-CD (6 h)	530–940	4.8	8.3	86.9	0.913
C-CD (8 h)	530–975	5.8	7	87.2	0.926

**Table 4:** Carbons functional surface groups determined by the Boehm method.

Carbon	Total Basic Groups (mmol g^−1^)	Total Acidic Groups (mmol g^−1^)	Total Functionalities (mmol g^−1^)	Phenolic Groups (mmol g^−1^)
C-CD (4 h)	0.312	0.249	0.561	0.249
C-CD (6 h)	0.359	0.186	0.545	0.186
C-CD (8 h)	0.312	0.412	0.724	0.412

**Table 5:** Kinetic parameters for the MB adsorption on the adsorbents.

Carbon	Temperature [K]	Pseudo-First-Order		Pseudo-Second-Order	
		*k*_1_ (min^−1^)	*R* ^2^	*k*_2_ (g mg^−1^ min^−1^)	*R* ^2^
C-CD (4 h)	298	0.0168	0.7889	0.00111	0.9999
	308	0.0052	0.7898	0.00011	0.9995
	315	0.0139	0.9329	0.00062	0.9995
C-CD (6 h)	298	0.0159	0.9386	0.00120	0.9999
	308	0.0154	0.9659	0.00102	0.9999
	315	0.0106	0.8338	0.00075	0.9997
C-CD (8 h)	298	0.0065	0.9312	0.00086	0.9995
	308	0.0041	0.9452	0.00092	0.9996
	315	0.0120	0.8604	0.00084	0.9997

**Table 6:** Kinetic parameters for the MG adsorption on the adsorbents.

Carbon	Temperature [K]	Pseudo-First-Order		Pseudo-Second-Order	
		*k*_1_ (min^−1^)	*R* ^2^	*k*_2_ (g mg^−1^ min^−1^)	*R* ^2^
C-CD (4 h)	298	0.0127	0.8857	0.00117	0.9995
	308	0.0187	0.9677	0.00096	0.9995
	315	0.0173	0.9724	0.00135	0.9999
C-CD (6 h)	298	0.0226	0.9736	0.00193	0.9999
	308	0.0203	0.9196	0.00166	0.9998
	315	0.0136	0.8564	0.00206	0.9999
C-CD (8 h)	298	0.0182	0.9690	0.00227	0.9999
	308	0.0163	0.9481	0.00350	1.0000
	315	0.0166	0.9362	0.00221	0.9999

**Table 7:** Intraparticle diffusion parameters for the MB adsorption.

Carbon	Temperature [K]	Parameter		
		*k_id_* (min^−1^)	*c*	*R* ^2^
C-CD (4 h)	298	4.88	85.02	0.9513
	308	3.43	87.52	0.9188
	315	3.62	84.13	0.9606
C-CD (6 h)	298	4.83	86.05	0.9644
	308	4.72	83.10	0.9811
	315	5.75	69.57	0.9627
C-CD (8 h)	298	2.35	111.26	0.9881
	308	2.48	100.08	0.9948
	315	3.38	94.27	0.9580

**Table 8:** Intraparticle diffusion parameters for the MG adsorption.

Carbon	Temperature [K]	Parameter		
		*k_id_* (min^−1^)	*c*	*R* ^2^
C-CD (4 h)	298	2.92	93.10	0.9381
	308	3.60	85.82	0.9703
	315	3.04	84.82	0.9782
C-CD (6 h)	298	2.79	100.83	0.9742
	308	3.11	95.85	0.9949
	315	2.21	95.79	0.9822
C-CD (8 h)	298	1.53	114.29	0.9956
	308	1.46	114.14	0.9949
	315	1.90	101.02	0.9662

**Table 9:** Parameters from the Langmuir and Freundlich adsorption isotherm models (MB adsorption).

Carbon	Isotherm	Parameter	Temperature		
			298 K	308 K	315 K
**C-CD (4 h)**	Langmuir	*q_m exp_* (mg g^−1^)	195	183	166
		*q_m_* (mg g^−1^)	196	185	167
		*K_L_* (dm^3^ mg^−1^)	0.0630	0.0566	0.0916
		*R* ^2^	0.9996	0.9989	0.9998
	Freundlich	*K_F_* (mg^1−1/*n*^ (dm^3^)^1/*n*^ g^−1^)	74.64	75.52	80.04
		*n*	6.51	7.24	8.66
		*R* ^2^	0.8188	0.7784	0.6797
**C-CD (6 h)**	*Langmuir*	*q_e exp_* (mg g^−1^)	232	207	186
		*q_m_* (mg g^−1^)	238	208	189
		*K_L_* (dm^3^ mg^−1^)	0.0437	0.0404	0.0567
		*R* ^2^	0.9992	0.9979	0.9991
	Freundlich	*K_F_* (mg^1−1/*n*^ (dm^3^)^1/*n*^ g^−1^)	68.96	69.63	74.39
		*n*	5.12	5.93	6.98
		*R* ^2^	0.9115	0.8564	0.7948
**C-CD (8 h)**	Langmuir	*q_m exp_* (mg g^−1^)	349	268	238
		*q_m_* (mg g^−1^)	357	278	244
		*K_L_* (dm^3^ mg^−1^)	0.0870	0.0336	0.0477
		*R* ^2^	0.9988	0.9985	0.9992
	Freundlich	*K_F_* (mg^1−1/*n*^ (dm^3^)^1/*n*^ g^−1^)	105.63	61.54	65.51
		*n*	4.80	4.18	4.67
		*R* ^2^	0.9688	0.9426	0.9058

**Table 10:** Parameters from the Langmuir and Freundlich adsorption isotherm models (MG adsorption).

Carbon	Isotherm	Parameter	Temperature		
			298 K	308 K	315 K
**C-CD (4 h)**	Langmuir	*q_m exp_* (mg g^−1^)	161	155	145
		*q_m_* (mg g^−1^)	164	156	147
		*K_L_* (dm^3^ mg^−1^)	0.0738	0.1026	0.0646
		*R* ^2^	0.9997	0.9999	0.9993
	Freundlich	*K_F_* (mg^1−1/*n*^ (dm^3^)^1/*n*^ g^−1^)	79.25	81.58	77.20
		*n*	8.95	9.81	10.27
		*R* ^2^	0.8756	0.8300	0.8975
**C-CD (6 h)**	Langmuir	*q_e exp_* (mg g^−1^)	206	194	150
		*q_m_* (mg g^−1^)	213	200	154
		*K_L_* (dm^3^ mg^−1^)	0.0457	0.0396	0.0429
		*R* ^2^	0.9997	0.9990	0.9984
	Freundlich	*K_F_* (mg^1−1/*n*^ (dm^3^)^1/*n*^ g^−1^)	67.11	68.45	70.36
		*n*	5.57	6.15	8.68
		*R* ^2^	0.9557	0.9696	0.9110
**C-CD (8 h)**	Langmuir	*q_m exp_* (mg g^−1^)	274	256	165
		*q_m_* (mg g^−1^)	286	263	167
		*K_L_* (dm^3^ mg^−1^)	0.0356	0.0286	0.0424
		*R* ^2^	0.9990	0.9988	0.9991
	Freundlich	*K_F_* (mg^1−1/*n*^ (dm^3^)^1/*n*^ g^−1^)	63.55	55.58	66.45
		*n*	4.19	4.08	7.18
		*R* ^2^	0.9681	0.9740	0.9401

**Table 11:** Free enthalpy (Δ*G*) values for the MB and MG adsorption on the adsorbents.

Carbon	Temperature	∆*G* (kJ/mol) MB	∆*G* (kJ/mol) MG
C-CD (4 h)	298	−24.56	−25.39
	308	−25.10	−27.09
	315	−26.94	−26.49
C-CD (6 h)	298	−23.65	−24.20
	308	−24.24	−24.65
	315	−25.68	−25.42
C-CD (8 h)	298	−25.35	−23.59
	308	−23.77	−23.82
	315	−25.23	−25.39

**Table 12:** Comparison of the characteristics of activated carbons with others.

Precursor	Carbon	S_BET_ m^2^ g^−1^	Activation Method	MB (mg g^−1^)	MG (mg g^−1^)	Ref.
CDs	C-CD (4 h)	399	CO_2_	195	161	This study
CDs	C-CD (6 h)	679	CO_2_	232	206	This study
CDs	C-CD (8 h)	1136	CO_2_	357	274	This study
PET bottles	AC	353.307	KOH	404	-	[27]
waste granulated PET	PET-2-700N	1334	KOH	368	-	[6]
PVC	APVC	2666	KOH	838	-	[1]
commercial	F400	1003	-	409	-	[1]
PET waste	graphene	721.7	-	867		[77]
PET waste	ACK	1390	K_2_CO_3_	625		[15]
commercial	AC	900	-	303	-	[15]
waste polystyrene foam (PF)	AC-800	2712	KOH	1042	-	[12]
commercially powdered activated carbon (Merk)	CPAC	-	-	-	222	[78]
commercial	Coconut-AC	1101	-	-	91	[79]
commercial	Coal-AC	923	-	-	83.5	[79]
commercial	Aprocot-AC	819	-	-	74	[79]
commercial	Peach-AC	793	-	-	74	[79]

## Data Availability

Data are contained within this article.

## References

[1] Lian F., Xing B., Zhu L. (2011). Comparative study on composition, structure, and adsorption behavior of activated carbons derived from different synthetic waste polymers. J. Colloid Interface Sci..

[2] Al-Salem S.M., Lettieri P., Baeyens J. (2009). Recycling and recovery routes of plastic solid waste (PSW): A review. Waste Manag..

[3] Bratek W., Świątkowski A., Pakuła M., Biniak S., Bystrzejewski M., Szmigielski R. (2013). Characteristics of activated carbon prepared from waste PET by carbon dioxide activation. J. Anal. Appl. Pyrolysis.

[4] Kaur B., Singh J., Gupta R.K., Bhunia H. (2019). Porous carbons derived from polyethylene terephthalate (PET) waste for CO_2_ capture studies. J. Environ. Manag..

[5] Mendoza-Carrasco R., Cuerda-Correa E.M., Alexandre-Franco M.F., Fernández-González C., Gómez-Serrano V. (2016). Preparation of high-quality activated carbon from polyethyleneterephthalate (PET) bottle waste. Its use in the removal of pollutants in aqueous solution. J. Environ. Manag..

[6] Cansado I.P.P., Galacho C., Nunes Â.S., Carrot M.L.R., Carrot P.J.M. (2010). Adsorption properties of activated carbons prepared from recycled PET in the removal of organic pollutants from aqueous solutions. Adsorpt. Sci. Technol..

[7] Marzec M., Tryba B., Kaleńczuk R.J., Morawski A.W. (1999). Poly(ethylene terephthalate) as a source for activated carbon. Polym. Adv. Technol..

[8] Parra J.B., Ania C.O., Arenillas A., Rubiera F., Pis J.J. (2004). High value carbon materials from PET recycling. Appl. Surf. Sci..

[9] Parra J.B., Ania C.O., Arenillas A., Pis J.J. (2002). Textural characterisation of activated carbons obtained from poly(ethylene terephthalate) by carbon dioxide activation. Stud. Surf. Sci. Catal..

[10] Esfandiari A., Kaghazchi T., Soleimani M. (2012). Preparation and evaluation of activated carbons obtained by physical activation of polyethyleneterephthalate (PET) wastes. J. Taiwan Inst. Chem. Eng..

[11] László K., Bóta A., Nagy L.G., Cabasso I. (1999). Porous carbon from polymer waste materials. Colloids Surf. A Physicochem. Eng. Asp..

[12] de Paula F.G.F., de Castro M.C.M., Ortega P.F.R., Blanco C., Lavall R.L., Santamaría R. (2018). High value activated carbons from waste polystyrene foams. Microporous Mesoporous Mater..

[13] Choma J., Osuchowski Ł., Jaroniec M. (2014). Properties and applications of activated carbons obtained from polymeric materials. Ochr. Sr..

[14] Choma J., Marszewski M., Osuchowski L., Jagiello J., Dziura A., Jaroniec M. (2015). Adsorption properties of activated carbons prepared from waste CDs and DVDs. ACS Sustain. Chem. Eng..

[15] Mantia F.P.L., Liarda D., Ceraulo M., Mistretta M.C. (2023). A Green approach for recycling compact discs. Polymers.

[16] Méndez-Liñán L., López-Garzón F.J., Domingo-García M., Pérez-Mendoza M. (2010). Carbon adsorbents from polycarbonate pyrolysis char residue: Hydrogen and methane storage capacities. Energy Fuels.

[17] de Castro C.S., Viau L.N., Andrade J.T., Mendonçac T.A.P., Gonçalvesc M. (2018). Mesoporous activated carbon from polyethyleneterephthalate (PET) waste: Pollutant adsorption in aqueous solution. New J. Chem..

[18] Cândido N.R., Prauchner M.J., de Oliveira Vilela A., Pasa V.M.D. (2020). The use of gases generated from eucalyptus carbonization as activating agent to produce activated carbon: An integrated process. J. Environ. Chem. Eng..

[19] Yi H., Nakabayashi K., Yoon S.-H., Miyawaki J. (2021). Pressurized physical activation: A simple production method for activated carbon with a highly developed pore structure. Carbon.

[20] Shahcheragh S.K., Bagheri Mohagheghi M.M., Shirpay A. (2023). Effect of physical and chemical activation methods on the structure, optical absorbance, band gap and urbach energy of porous activated carbon. SN Appl. Sci..

[21] Mazlan M.A.F., Uemura Y., Yusup S., Elhassan F., Uddin A., Hiwada A., Demiya M. (2016). Activated carbon from rubber wood sawdust by carbon dioxide activation. Procedia Eng..

[22] Khalili S., Khoshandam B., Jahanshahi M. (2016). A comparative study of CO_2_ and CH_4_ adsorption using activated carbon prepared from pine cone by phosphoric acid activation. Korean J. Chem. Eng..

[23] Oginni O., Singh K., Oporto G., Dawson-Andoh B., McDonald L., Sabolsky E. (2019). Influence of one-step and two-step KOH activation on activated carbon characteristics. Bioresour. Technol. Rep..

[24] Dotto G.L., Santos J.M.N., Rodrigues I.L., Rosa R., Pavan F.A., Lima E.C. (2015). Adsorption of methylene blue by ultrasonic surface modified chitin. J. Colloid Interface Sci..

[25] Bergna D., Hu T., Prokkola H., Romar H., Lassi U. (2020). Effect of some process parameters on the main properties of activated carbon produced from peat in a lab-scale process. Waste Biomass Valoriz..

[26] Wiśniewska M., Marciniak M., Gęca M., Herda K., Pietrzak R., Nowicki P. (2022). Activated biocarbons obtained from plant biomass as adsorbents of heavy metal ions. Materials.

[27] Bazan-Wozniak A., Wolski R., Paluch D., Nowicki P., Pietrzak R. (2022). Removal of organic dyes from aqueous solutions by activated carbons prepared from residue of supercritical extraction of marigold. Materials.

[28] Din S.F.M., Othman N., Mohamad Z., Man S.H.C., Karim K.J.A., Hassan A. (2020). Recycled poly(ethylene terephthalate) as dye adsorbent: A mini-review. Chem. Eng. Trans..

[29] Djahed B., Shahsavani E., Naji F.K., Mahvi A.H. (2016). A novel and inexpensive method for producing activated carbon from waste polyethylene terephthalate bottles and using it to remove methylene blue dye from aqueous solution. Desalin. Water Treat..

[30] Oladoye P.O., Ajiboye T.O., Omotola E.O., Oyewola O.J. (2022). Methylene blue dye: Toxicity and potential elimination technology from wastewater. Results Eng..

[31] Ojediran J.O., Dada A.O., Aniyi S.O., David R.O., Adewumi A.D. (2021). Mechanism and isotherm modeling of effective adsorption of malachite green as endocrine disruptive dye using Acid Functionalized Maize Cob (AFMC). Sci. Rep..

[32] Srivastava S., Sinha R., Roy D. (2004). Toxicological effects of malachite green. Aquat. Toxicol..

[33] Mukherjee T., Das M. (2013). Degradation of malachite green by enterobacter asburiae Strain XJUHX-4TM. Clean-Soil Air Water.

[34] Brunauer S., Emmett P.H., Teller E. (1938). Adsorption of gases in multimolecular layers. J. Am. Chem. Soc..

[35] Kruk M., Jaroniec M. (2001). Gas Adsorption characterization of ordered organic-inorganic nanocomposite materials. Chem. Mater..

[36] Jagiello J., Olivier J.P. (2013). 2D-NLDFT Adsorption models for carbon slit-shaped pores with surface energetical heterogeneity and geometrical corrugation. Carbon.

[37] Jagiello J., Olivier J.P. (2013). Carbon slit pore model incorporating surface energetical heterogeneity and geometrical corrugation. Adsorption.

[38] Jedynak K., Charmas B. (2023). Adsorption properties of biochars obtained by KOH activation. Adsorption.

[39] Pereira R.C., Kaal J., Arbestain M.C., Lorenzo R.P., Aitkenhead W., Hedley M., Macías F., Hindmarsh J., Maciá-Agulló J.A. (2011). Contribution to characterisation of biochar to estimate the labile fraction of carbon. Org. Geochem..

[40] Cárdenas-Aguiar E., Gascó G., Paz-Ferreiro J., Méndez A. (2019). Thermogravimetric analysis and carbon stability of chars produced from slow pyrolysis and hydrothermal carbonization of manure waste. J. Anal. Appl. Pyrol..

[41] Boehm H.P. (1994). Some aspects of the surface chemistry of carbon blacks and other carbons. Carbon.

[42] Boehm H.P. (2002). Surface oxides on carbon and their analysis: A critical assessment. Carbon.

[43] Lim C.K., Bay H.H., Noeh C.H., Aris A., Majid Z.A., Ibrahim Z. (2013). Application of zeolite-activated carbon macrocomposite for the adsorption of Acid Orange 7: Isotherm. kinetic and thermodynamic studies. Environ. Sci. Pollut. Res..

[44] Rivera-Utrilla J., Bautista-Toledo I., Ferro-García M.A., Moreno-Castilla C. (2001). Activated carbon surface modifications by adsorption of bacteria and their effect on aqueous lead adsorption. J. Chem. Technol. Biotechnol..

[45] Jedynak K., Charmas B. (2023). Activated biocarbons obtained from lignocellulosic precursors as potential adsorbents of ammonia. Physicochem. Probl. Miner. Process..

[46] Lagergren S. (1898). About the theory of so-called adsorption of soluble substances. Kungl. Sven. Veten. Akad. Handl..

[47] Ho Y.S., McKay G. (1999). Pseudo-second-order model for sorption processes. Process Biochem..

[48] Weber W.J., Morris J.C. (1963). Kinetics of adsorption on carbon solution. J. Sanit. Eng. Div. Am. Soc. Civ. Eng..

[49] Langmuir I. (1916). The constitution and fundamental properties of solids and liquids. Part I. Solids. J. Am. Chem. Soc..

[50] Freundlich H.M.F. (1906). Over the adsorption in solution. J. Phys. Chem..

[51] Freundlich H. (1907). Über die Adsorption in Lösungen. Z. Phys. Chem..

[52] Kurdziel K., Raczyńska-Żak M., Dąbek L. (2019). Equilibrium and kinetic studies on the process of removing chromium(VI) from solutions using HDTMA-modified halloysite. Desalin. Water Treat..

[53] Sing K.S.W., Everett D.H., Haul R.A.W., Moscou L., Pierotti R.A., Rouquerol J., Siemieniewska T. (1985). Reporting physisorption data for gas/solid systems with special reference to the determination of surface area and porosity. Pure Appl. Chem..

[54] Choma J. (2006). Characterization of nanoporous active carbons by using gas adsorption isotherms. Wegiel Aktywny w Ochronie Srodowiska i Przemysle.

[55] Everett D.H. (1972). IUPAC, Manual of symbol and terminology for physicochemical quantities and units, appendix, Definitions, terminology and symbols in Colloid and Surface Chemistry, Part I. Pure Appl. Chem..

[56] Ferrari A.C., Robertson J. (2000). Interpretation of Raman spectra of disordered and amorphous carbon. Phys. Rev. B.

[57] Blankenship L.S., Balahmar N., Mokaya R. (2017). Oxygen-rich microporous carbons with exceptional hydrogen storage capacity. Nat. Commun..

[58] Vaughn S.F., Kenar J.A., Thompson A.R., Peterson S.C. (2013). Comparison of biochars derived from wood pellets and pelletized wheat straw as replacements for peat in potting substrates. Ind. Crops Prod..

[59] Ma R., Ma Y., Gao Y., Cao J. (2020). Preparation of micro-mesoporous carbon from seawater-impregnated sawdust by low temperature one-step CO_2_ activation for adsorption of oxytetracycline. SN Appl. Sci..

[60] Marciniak M., Goscianska J., Pietrzak R. (2018). Physicochemical characterization of ordered mesoporous carbons functionalized by wet oxidation. J. Mater. Sci..

[61] Sen T.K., Afroze S., Ang H.M. (2011). Equilibrium, kinetics and mechanism of removal of methylene blue from aqueous solution by adsorption onto pine cone biomass of Pinus radiata. Water Air Soil Pollut..

[62] Garnuszek M., Szczepanik B., Gawinkowski S., Słomkiewicz P.M., Witkiewicz Z., Jedynak K. (2012). Spectral characterization of mesoporous carbons modified by Ag, Au, TiO_2_ and Fe_3_O_4_ nanoparticles. Ochr. Śr..

[63] Kim D.J., Lee H.I., Yie J.E., Kim S.-J., Kim J.M. (2005). Ordered mesoporous carbons: Implication of surface chemistry, pore structure and adsorption of methyl mercaptan. Carbon.

[64] Emrooz H.B.M., Maleki M., Rashidi A., Shokouhimehr M. (2021). Adsorption mechanism of a cationic dye on a biomass-derived microand mesoporous carbon: Structural, kinetic, and equilibrium insight. Biomass Convers. Biorefin..

[65] Jedynak K., Charmas B. (2021). Preparation and characterization of physicochemical properties of spruce cone biochars activated by CO_2_. Materials.

[66] Figueiredo J.L., Pereira M.F.R., Freitas M.M.A., Órfao J.J.M. (1999). Modification of the surface chemistry of activated carbons. Carbon.

[67] Donia A.M., Yousif A.M., Atia A.A., Elsamalehy M.F. (2014). Efficient adsorption of Ag(I) and Au(III) on modified magnetic chitosan with amine functionalities. Desalin. Water Treat..

[68] Radnia H., Ghoreyshi A.A., Younesi H., Najafpour G.D. (2012). Adsorption of Fe(II) ions from aqueous phase by chitosan adsorbent: Equilibrium, kinetic, and thermodynamic studies. Desalin. Water Treat..

[69] Belhachemi M., Belala Z., Lahcene D., Addoun F. (2009). Adsorption of phenol and dye from aqueous solution using chemically modified date pits activated carbons. Desalin. Water Treat..

[70] Sahoo T.R., Prelot B., Bonelli B., Freyria F.S., Rossetti I., Sethi R. (2020). Adsorption processes for the removal of contaminants from wastewater: The perspective role of nanomaterials and nanotechnology. Nanomaterials for the Detection and Removal of Wastewater Pollutants.

[71] Charmas B., Zięzio M., Jedynak K. (2023). Assessment of the porous structure and surface chemistry of activated biocarbons used for methylene blue adsorption. Molecules.

[72] Jedynak K., Repelewicz M., Kurdziel K., Wideł D. (2021). Mesoporous carbons as adsorbents to removal of methyl orange (anionic dye) and methylene blue (cationic dye) from aqueous solutions. Desalin. Water Treat..

[73] Worch E. (2012). Adsorption Technology in Water Treatment: Fundamentals, Processes and Modeling.

[74] Duong D.D. (1998). Adsorption Analysis: Equilibrum and Kinetics.

[75] Milenković D.D., Milosavljević M.M., Marinković A.D., Ðokić V.R., Mitrović J.Z., Bojić A.L. (2013). Removal of copper(II) ion from aqueous solution by high-porosity activated carbon. Water Sa.

[76] Sahu S., Pahi S., Tripathy S., Singh S.K., Behera A., Sahu U.K., Patel R.K. (2020). Adsorption of methylene blue on chemically modified lychee seed biochar: Dynamic, equilibrium, and thermodynamic study. J. Mol. Liq..

[77] Essawy N.A.E., Ali S.M., Farag H.A., Konsowa A.H., Elnouby M., Hamad H.A. (2017). Green synthesis of graphene from recycled PET bottle wastes for use in the adsorption of dyes in aqueous solution. Ecotoxicol. Environ. Saf..

[78] Malik R., Ramteke D.S., Wate S.R. (2007). Adsorption of malachite green on groundnut shell waste based powdered activated carbon. Waste Manag..

[79] Qu Q.W., Yuan T., Yin G., Xu S., Zhang Q., Su H. (2019). Effect of properties of activated carbon on malachite green adsorption. Fuel.

